# Almond Hull as a Functional Ingredient of Bread: Effects on Physico-Chemical, Nutritional, and Consumer Acceptability Properties

**DOI:** 10.3390/foods11060777

**Published:** 2022-03-08

**Authors:** Maher Kahlaoui, Marta Bertolino, Letricia Barbosa-Pereira, Hayet Ben Haj Kbaier, Nabiha Bouzouita, Giuseppe Zeppa

**Affiliations:** 1 Department of Agriculture, Forest and Food Sciences (DISAFA), University of Turin, 10095 Grugliasco, Italy; maherkahlaoui1@gmail.com (M.K.); marta.bertolino@unito.it (M.B.); letricia.barbosa.pereira@usc.es (L.B.-P.); 2 Innovation and Valorisation Laboratory for a Sustainable Food Industry, Higher School of Food Industries, Tunis 1003, Tunisia; h.kbaier@gmail.com (H.B.H.K.); bouzouita.nabiha@gmail.com (N.B.); 3 Department of Analytical Chemistry, Nutrition and Food Science, Faculty of Pharmacy, University of Santiago de Compostela, 15705 Santiago de Compostela, Spain; 4 Laboratory of Structural Organic Chemistry, Synthesis and Physico-Chemical Analysis, University of Tunis El-Manar, Tunis 1068, Tunisia

**Keywords:** almond hull, polyphenols, antioxidant capacity, liking test, gastrointestinal digestion

## Abstract

Hulls are the principal almond by-products and are rich in bioactive compounds, such as polyphenols and fibre. Generally, hulls are used as animal feed; however, because of their valuable chemical composition, alternative applications as a natural food ingredient and dietary supplement should be evaluated. The aim of this study was to assess the physico-chemical and nutritional characteristics and the consumer acceptability of bread produced by replacing 4% and 8% of wheat flour with almond hulls (AHs) obtained from six almond varieties at two ripening stages (green and mature). The use of AHs in bread production increased fibre content, polyphenol content, and antioxidant activity. In particular, bread containing mature AHs showed the highest quantities of fibre and sugars, mainly glucose, whereas bread containing green AHs showed the highest polyphenol content. The polyphenol content and antioxidant activity in bread containing green AHs were 272.88 mg GAE/100 g dry weight and 1145.32 μmol TE/100 g dry weight, respectively, of which 60.5% and 52% were bioaccessible after in vitro digestion. Bread containing AH powder showed slightly lower specific volume, darker crumb colour, and lower hardness than those of the control. Consumer evaluation indicated that breads with 8% AH powder were those with the most overall liking.

## 1. Introduction

The almond tree (*Prunus dulcis* (Mill.) D.A. Webb, *Prunus amygdalus* Batch, or *Amygdalus communis* L.) is one of the most popular nut trees worldwide and ranks number-one in nut production with over 3 million tonnes of almond fruits yearly produced throughout an area of over 2 million ha [1]. The kernel, the edible part of the almond, is a seed with two large cotyledons; it is consumed worldwide unblanched (with the skin) or blanched (without the skin) raw, cooked, or dry-roasted, and whole, sliced, or ground. It is extensively consumed as a snack or used in food preparation, especially in confectioneries, bakeries, and chocolates, as well as in pharmaceutical and cosmetic applications.

Almond kernel production generates large amounts of by-products, among which the main one is the hull, accounting for 35–62% of the total fresh weight of the almond [2] with an annual production of more than six million tonnes [2,3]. Consequently, novel solutions are required to add value to these residues, with the aim of improving the economic profit and environmental sustainability of large-scale almond production. Generally, the almond hull is dry and obtained during the harvesting of mature almonds. In some countries such as Tunisia, almonds have also been harvested green in order to produce a special almond oil. In these countries, there is also a production of green hull removed before the oil extraction.

The sugar content in almond hulls reportedly ranges from 18.0 to 30.0%, protein content varies from 2.1 to 8.8%, and crude fibre ranges from 10.0 to 24.9% [2]. Acid detergent fibre varies from 20.6 to 35.2%, neutral detergent fibre from 10.0 to 15.0%, cellulose from 20.6 to 35.2%, and crude lignin ranges from 7.5 to 15.6% [2]. Generally, this by-product is used as livestock feed or fuel material [2], but it is a rich source of triterpenoids (betulinic, urosolic, and oleanolic acids), flavonol glycosides, phenolic acids (caffeic, ferulic, *p*-coumaric, and synaptic acids), catechin, protocatechuic acid, vanillic acid, and other polyphenolic compounds; therefore, it may be an interesting source of natural antioxidants and other bioactive compounds [3,4,5,6,7]. The total polyphenolic content is comprised of between 35.9 and 166.7 mg GAE/g extract [4,6]. When incorporated into the diet, almond hulls (AHs) not only reduce colon cancer risk in rats, increase high-density lipoprotein cholesterol levels, and reduce LDL cholesterol levels in humans [4,6], but also reduce DNA scission and metal ion chelation activities [4,5].

Recently, AHs were incorporated into feedstocks for cow, hens, and edible larvae [4]. The obtained results showed that there are no effects on milk and egg composition quality, while for edible larvae there is an increase in weight, yield, and calcium content [4]. In addition, Takeoka and Dao [8] evaluated the use of AH as a natural source of sweetener concentrate and dietary fibre. On the contrary, to our knowledge, no information is available in the literature describing the direct use of AH as a food ingredient. Therefore, the aim of this research was to evaluate the effect on the physico-chemical characteristics and consumer acceptability of the use of different concentrations of green and mature AHs as an ingredient in bread to valorise its polyphenol and fibre content. The breads thus obtained were compared with those acquired using only wheat flour and those fortified with wheat bran to evaluate whether the use of AH could promote better results in comparison with the commonly applied functionalization.

## 2. Materials and Methods 

### 2.1. Chemicals 

Folin–Ciocalteu phenol reagent (2 M), 2,2-diphenyl-1-picrylhydrazyl (DPPH), 6-hydroxy-2,5,7,8-tetramethylchroman-2-carboxylic acid (97%; Trolox), sodium carbonate (≥99.5%), methanol (≥99.9%), formic acid (98–100%), gallic acid (≥98%), ethanol (≥99.9%), sodium hydroxide (1 M), α-amylase from *Bacillus* sp., pepsin from porcine gastric mucosa, pancreatin from porcine pancreas, and bile salts were obtained from Sigma-Aldrich (Milan, Italy). 

Potassium phosphate dibasic, potassium phosphate monobasic, potassium chloride, sodium bicarbonate, sodium chloride, magnesium chloride hexahydrate, ammonium carbonate, hydrochloric acid (fuming 37%), and calcium chloride dihydrate were provided by Carlo Erba (Milan, Italy). Ultrapure water was prepared using a Milli-Q filter system (Millipore, Milan, Italy).

### 2.2. Materials

For this study, AHs from green and mature almonds of three Italian varieties (Fascionello, Pizzuta, and Romana) provided by the Consorzio della Mandorla d’Avola, Italy and three Tunisian varieties (Achaak, Fakhfekh, and Laurane) provided by the Tunisian Office of almond and olive oils (Sfax, Tunisia) were used. 

The green AHs were separated manually and then dried in an oven UFE 550 model (Memmert, Schwabach, Germany) for 24 h at 40 °C. All the AHs were ground using a Retsch ZM200 grinder (Retsch Gmbh, Haan, Germany) and sieved to obtain powders with particle sizes between 100 and 250 μm. The powders were stored in vacuum-sealed polyethylene bags at 4 °C until further use.

Wheat bran of the Aubusson variety was obtained using a laboratory-scale mill Labormill 4 RB (Bona, Monza, Italy) and sieved to obtain powders with particle sizes between 100 and 250 μm.

Ingredients for bread production, such as wheat flour (carbohydrate 72.5 g/100 g, protein 10 g/100 g, fibre 2.5 g/100 g, and fat 2 g/100 g), dried yeast, sodium chloride, saccharose, and water were purchased from a local retailer (Carrefour, Grugliasco, Torino, Italy).

### 2.3. Bread Production

For bread production, a home bread machine Moulinex OW6101 (SEB Italia, Milan, Italy) was used and programmed to include 40 min of kneading, 80 min of fermentation at 30 °C, and 60 min of baking at 180 °C. AH powders and wheat bran were used as wheat flour replacers at 4% and 8% (*w*/*w*), respectively (Table 1). These additions were defined according to preliminary tests (data not shown). The baked loafs were cooled at ambient temperature for 2 h, sliced (20 mm-thick), and stored in vacuum-sealed polyethylene bags at 4 °C for chemical analysis. All productions were performed in duplicate.

### 2.4. Physico-Chemical Analysis of Powders

Dry matter content was determined at 105 °C using a Gibertini Eurotherm electronic moisture balance (Gibertini Elettronica, Novate Milanese, Milan, Italy) with 5 g of powder.

Ash was obtained after mineralisation of the samples in a muffle furnace at 550 °C for 6 h, according to the method described by Baldini et al. [9].

Protein content was calculated multiplying the nitrogen content determined with the Kjeldahl method by 6.25. Fat content was determined using a Soxhlet extraction apparatus with petroleum ether as the solvent for 6 h. Total, insoluble, and soluble fibre contents were determined according to the AOAC method 991.43 [10]. Carbohydrates were estimated as the difference.

Sugars and organic acids of AHs were determined using liquid chromatography, according to Turki et al. [11]. The AH powder (1 g) was added to 10 mL of ultra-pure water, treated for 10 min in an ultrasonic bath Sonorex Digitec DT 103 H (VWR, Milan, Italy), and then centrifuged for 10 min at 10,000× *g* at 10 °C using an MPW-380R centrifuge (MPW, Warsaw, Poland). The supernatant was filtered through a 0.45 µm polypropylene membrane filter and stored at −18 °C until analysis.

The high-performance liquid chromatography system (Thermo Finningan, San Jose, CA, USA) was equipped with a gradient pump (P4000), multiple autosampler (AS3000) fitted with a 20 µL loop, UV detector (UV100) set at 210 and 290 nm, and refractive index detector RI-150. Data were collected using ChromQuest 3.0 (Thermo Finningan). The analyses were performed isocratically at 0.8 mL/min and 65 °C with a 300 × 7.8 mm internal diameter cation exchange column (Aminex HPX-87H) equipped with a cation H+ microguard cartridge (Bio-Rad Laboratories, Hercules, CA, USA). The mobile phase used was 0.013 N H_2_SO_4_. Identification was achieved by comparing with the retention times of the authentic standards.

The oil-binding capacity (OBC) was determined according to Femenia et al. [12], with minor modifications. Powder samples (0.5 g) were mixed with olive oil (10 mL), left overnight at 20 °C, centrifuged at 1500× *g* for 5 min, and the excess oil was discarded. The samples were weighed and the OBC was expressed as g oil/g dry powder. The water-holding capacity (WHC) was determined according to Sudha et al. [13]. Powder samples (1 g) were mixed with 50 mL of distilled water, centrifuged at 1000× *g* for 15 min, and the excess water was discarded. The samples were weighed and the WHC was expressed as g water/g dry powder. The swelling capacity (SWC) was evaluated according to the method described by Femenia et al. [12], with slight modifications. Dried powder samples (0.5 g) were vigorously mixed with 10 mL of distilled water and left overnight at 20 °C to allow the fibre to swell. The SWC was measured as the bed volume after equilibration in excess solvent and expressed as mL/g dry powder.

### 2.5. Physico-Chemical Analysis of Breads 

Water activity (a_w_) was determined at 25 ± 0.02 °C using an Aqua-Lab CX-2T (Decagon Devices, Pullman, WA, USA). 

The bread crumb colour was evaluated using a CM-5 spectrophotometer (Konica Minolta, Tokyo, Japan) in transmittance mode. CIELAB parameters were used to measure the bread colour, where L* is the lightness ranging from 0 (black) to 100 (white), a* indicates the colours from red-purple (positive a*) to bluish-green (negative a*), and b* denotes the colours from yellow (positive b*) to blue (negative b*). The ΔE* parameter, which represents the difference between two colours and is perceptible by the human eye when >2.5, was calculated as follows [14]:

ΔE* = [(L_2_* −L_1_*)^2^ + (a_2_* − a_1_*)^2^ + (b_2_* − b_1_*)^2^]^1/2^ (1)

Loaf volume was determined 2 h after baking using the rapeseed displacement standard method 10-05 AACC [15].

Images of four bread slices were acquired using a Scanjet 5590 (HP, Milan, Italy) and saved as bitmap files at a resolution of 1200 dpi in the RGB colour space. From the images, a single square (50 mm × 50 mm) was drawn, converted to an 8-bit grey scale, and binarised. The mean cell area (mm^2^), cell density (cells/mm^2^), circularity, percentage of the area occupied by cells, and percentage of the cell distribution were measured using the ImageJ1.53k software package (https://imagej.nih.gov/ij/; accessed on 20 September 2021) according to Scheuer et al. [16].

Textural analyses were performed on four bread slices (20 mm-thick) 2 h after the baking phase. The TPA test was performed using a TA.XT2i Plus Texture Analyser^®^ (Stable Micro System, Godalming, UK) equipped with a 25 kg load cell and an SMS P/100 probe. The slices were compressed in the central area for 60% height deformation with a waiting time before the second bite of 30 s and using a speed test of 1 mm/s. The trigger force was set at 0.02 kg.

For the acquisition of the force–time curve, Texture Expert Exceed software 2.54 (Stable Micro System, Godalming, UK) was used. The parameters analysed were hardness (N), cohesiveness (dimensional), adhesiveness (mJ), gumminess (N), and springiness (mm). 

### 2.6. Polyphenol Extraction

Polyphenols were extracted according to the method described by Guglielmetti et al. [17], with slight modifications. Briefly, 0.5 g of the AH powder or 1 g of freeze-dried bread obtained with an LIO-5P DIGITAL Freeze Dryer (Bioclass S.r.l., Pistoia, Italy) working at 50 mbar for 48 h was mixed with 10 mL of ethanol/water solution (50/50, *v*/*v*). Extractions were performed at 25 °C for 2 h with a VDRL 711 orbital shaker (Asal S.r.l., Milan, Italy) under constant rotatory agitation at 60 rpm. All extracts were centrifuged at 2800× *g* for 10 min at 4 °C, and the supernatants were collected and filtered through a 0.45 µm nylon membrane filter. The samples were stored in amber vials at –18 °C before analysis. All extractions were performed in triplicate.

### 2.7. Total Phenolic Content

The total phenolic content (TPC) of the extract was determined according to the Folin–Ciocalteu colorimetric method adapted to a 96-well microplate using a spectrophotometric multi-detection microplate reader BioTek Synergy HT (BioTek Instruments, Milan, Italy) as described by Barbosa-Pereira et al. [18]. The absorbance was recorded at 740 nm and determined in triplicate.

A calibration curve of gallic acid (20–100 mg/L; R^2^ = 0.998) was used to quantify the phenolic content, which was expressed in milligrams of gallic acid equivalents per gram of dry powder (mg GAE/g DW).

### 2.8. Antioxidant Capacity

The antioxidant capacity of the extracts was determined with the DPPH* radical scavenging method following the procedure described by Barbosa-Pereira et al. [18]. The decrease in DPPH absorbance was measured at 517 nm using a spectrophotometric multi-detection microplate reader BioTek Synergy HT (BioTek Instruments, Milan, Italy). All assays were conducted in triplicate. The antioxidant capacity was calculated as the inhibition percentage (IP) of the DPPH radical as follows:

IP (%) = [(A_0_ − A_30_)/A_0_] × 100 (2)

where A_0_ is the absorbance of the blank and A_30_ is the absorbance at 30 min. 

A standard curve of Trolox (12.5–300 μM; R^2^ = 0.990) was used to determine the radical-scavenging activity (RSA), and the results were expressed as micromoles of Trolox equivalent per gram of dry powder (μmol TE/g DW).

### 2.9. Preliminary Consumer Acceptance Test 

The sensory test was conducted with 40 adult subjects (females = 70%, age range: 25–60 years) who were recruited from the staff of the University of Turin. Written informed consent was obtained from all participants before the test. Participants received individual trays with eight bread slices and rinsed their mouths with noncarbonated water before beginning the evaluation. Participants tasted the samples according to the tray presentation order, blind, and without any information about the innovativeness of the bread to avoid a potential effect of the information on the liking scores. Participants rated their liking for colour, appearance, odour, taste, flavour, and texture, and overall liking using a 9-point hedonic scale (1 = extremely dislike, 9 = extremely like) [19]. Breads were served in a randomized and balanced order. Participants were required to rinse their mouths with still water for approximately 1 min between the samples. Consumers took 10–15 min to complete the evaluation. The tests were performed in an air-conditioned room with white light at approximately 21 °C.

### 2.10. In Vitro Simulated Gastrointestinal Digestion (GID)

The digestion of bread samples was conducted using a three-phase (oral, gastric, and intestinal) standardized protocol according to Minekus et al. [20]. Briefly, 1 g of each freeze-dried bread was mixed with simulated digestive fluids (simulated salivary, gastric, and intestinal fluids) consisting of the corresponding electrolyte stock solutions, enzymes, and water. The electrolyte stock solutions were heated in an SW-20 water bath (Julabo GmbH, Seelbach, Germany) at 37 °C. The digestion process was repeated three times for each bread. A control, in which the sample was replaced with ultrapure water, was also prepared in triplicate to assess the contribution of digestion enzymes and simulated fluids in the subsequent analysis. Once the digestive phase was completed, the pH was lowered to 5.4 to stop the process. The samples were centrifuged at 12,500× *g* for 10 min at 4 °C, and the supernatants were filtered through 0.45 μm cellulose acetate filters.

The filtered samples were stored at −18 °C until subsequent analyses. In vitro bio-accessibility was calculated as follows:

% Bio-accessibility = CPOST/CPRE × 100(3)

where CPOST and CPRE correspond to the TPC values before and after the digestion process, respectively.

### 2.11. Statistical Analysis

The results were statistically analysed using the Statistica 13.3 software (StatSoft Inc., Tulsa, OK, USA). Physico-chemical data were subjected to one-way analysis of variance with Duncan’s post hoc test (95% confidence level). The Kruskal–Wallis H-test (95% confidence level) with a multiple comparison test was used to evaluate consumer acceptance.

## 3. Results

### 3.1. Physico-Chemical Characteristics of AH Powders

The chemical composition of the green and mature AHs used as flour replacers are listed in Table 2. 

The water content ranged from 26.37 to 31.02% for green materials and from 6.01 to 8.97% for mature materials. For both types of samples, values were significantly different (*p* < 0.05), and this difference may be due to the harvesting time, amount of rainfall, and the nature of the soil [2].

The ash content ranged from 6.98 to 10.45% for the green Fascionello and Romana varieties, respectively, and from 7.46 to 12.07% for the Pizzuta and Fakhfekh mature samples, respectively. Except for the Fakhfekh mature sample, these values are comparable with those determined by Prgomet et al. [2], who reported percentages ranging between 7.0 and 8.3%.

The high ash content of the AH powder indicates that it may be a good source of dietary minerals, and, depending on the harvesting time, it can be higher than 9.0% [21].

AHs have low fat content, ranging from 1.15 to 2.71%. Significant differences were observed between the green and mature samples, and the green varieties contained a significantly lower amount than those observed for mature varieties. The highest lipid content was recorded for the mature Romana variety (2.71 g/100 g DW), and the lowest was attributed to the green Laurane variety (1.15 g/100 g DW). These values were lower than those reported by Saura-Calixto et al. [22] for AHs (3.34%).

Nevertheless, the lipid content of mature samples was comparable to the values determined by Esfahlan et al. [23] for three AHs from Iranian cultivars, which ranged from 2.3 to 5.7%.

Regarding protein content, the highest concentrations were observed in the green varieties, except for the Achaak and Laurane varieties. The protein content ranged from 2.75 to 4.69% for green varieties and from 2.81 to 3.75% for the mature samples. These values were lower than those reported by Prgomet et al. [2], which ranged from 2.1 to 8.8%, but were similar to those reported by Esfahlan et al. [23], where the protein content ranged from 1.2 to 4.5% for AHs obtained from 40 cultivars.

AHs are characterized by a high content of carbohydrates, which are the main components. The results showed significant differences (*p* < 0.05) between the AH powders studied, and the highest concentrations were observed in the mature varieties. This difference can also be attributed to the origin of the variety, harvesting time, changes in agricultural approaches, and environmental conditions [24].

The total carbohydrate content varied between 41.72% and 56.28% for the mature samples of the Romana and Fakhfekh varieties, respectively, whereas for the green varieties, the highest concentration was observed in the Pizzuta variety (42.85%) and the lowest was observed in the Laurane variety (40.55%). These percentages were, in most cases, even higher than those found by Homedes et al. [21] and Saura-Calixto et al. [22], who reported that the carbohydrate content in AH ranges from 18.0 to 30.0%. 

Glucose was the major sugar identified in all the samples. Mature hull of the Achaak variety showed the highest concentration of fermentable sugars (204.38 mg/g DW) with 148.11 mg/g DW of glucose and 56.27 mg/g DW of fructose, whereas for xylose and fructose, the highest concentration was obtained with the Fascionello variety. These concentrations were lower than those reported by Holtman et al. [24] for Nonpareil AH (16.3% glucose, 15.9% fructose, and 5.2% saccharose) with 46.3% DW of total carbohydrate concentrations.

As for the acidic content, tartaric acid was the most abundant organic acid present in AH, especially for the mature Pizzuta (128.35 mg/g DW) and Fakhfekh (104.9 mg/g DW) samples. Nevertheless, the lowest content was observed in the mature Fascionello sample (55.25 mg/g DW). For all tested samples, the green varieties contained a significantly higher concentration of organic acids than the mature varieties. The highest concentration of malic acid was found for the Achaak variety (79.38 mg/g DW), while the lowest content was shown in the mature Laurane variety (16.61 mg/g DW) and green Fascionello sample (18.20 mg/g DW). The highest citric acid concentration was identified in the mature Laurane (15.58 mg/g DW) and green samples (11.55 mg/g DW). As for the fibre content (soluble and insoluble), significant differences were found among the different varieties. Except for the Pizzuta variety, the highest percentages were observed in the mature samples (*p* < 0.05). The total fibre content ranged from 23.32 to 34.93% for the green samples and from 25.32 to 35.77% for the mature samples. Apparently, the crude fibre content observed in these six varieties was higher than that previously reported (10.0–24.9%) [2]. 

The insoluble fibre concentration of AH powders in this study was comparable to that determined by Prgomet et al. [2] for AHs from 40 Iranian cultivars, which varied from 20.6 to 35.2%. Nevertheless, insoluble fibre contents were lower than those reported by Holtman et al. [24] for AH powders, which ranged from 10.0 to 15.0%. 

The results of the WHC, OBC, and SWC determinations are presented in Table 3. The WHC concentration ranged from 0.79 ± 0.03 g/g for wheat flour to a mean value of 4.39 ± 0.43 g/g for AH powders and 2.41 ± 0.40 for wheat bran, underlining strong relation of this parameter with the fibre content. The WHCs of the powders obtained from green hulls were higher (10–15%) than those of the powder from mature hulls; Fascionello and Fakhfekh varieties showed the highest WHC values for both vegetative stages. 

The OBC showed lesser differences between the almond varieties and hull vegetative stages, although, in this case, powders obtained from green hulls had higher OBC than those obtained from mature hulls. This may be related to the increase in the hydrophobic groups present in the cell wall through hull maturation or due to a change in the porosity of the structure that determines the entrapment of the oil in the system.

The SWC for wheat flour was lower (approximately 3.5 mL/g DW) than that for bran flour (approximately 4.6 mL/g DW) and all hull powder. Among almond varieties, Romana and Laurane were characterised by the highest capacity in the green vegetative state (6.58 ± 0.31 and 5.96 ± 0.27 mL/g DW, respectively), whereas in the mature state, the powders obtained from Fascionello and Achaak varieties showed the highest SWC concentrations (7.83 ± 0.72 and 6.73 ± 0.77 mL/g DW, respectively).

The WHC and SWC are important parameters to be considered in the production of functional foods because they underline the capability of the fibre to slow gastric emptying and bowel transit [12].

The TPC and RSA values showed significant differences among the almond varieties and origins, although the powders obtained from green hulls displayed higher values (20–25%) than those obtained from mature hulls (Table 4). Then, during the hull maturation, there is a reduction in polyphenols and antioxidant activity. Hull powders showed the highest TPC and RSA concentrations, which were approximately 30-fold higher than those observed for wheat bran flour. Fakhfekh green powder showed the highest TPC concentration (184.53 mg GAE/g DW), whereas refined wheat flour showed the lowest (1.13 mg GAE/g DW). Moreover, among almond varieties, the powder obtained from Pizzuta and Fakhfekh varieties presented the highest TPC concentrations (160.55 and 184.53 mg GAE/g DW, respectively, for green hulls and 153.11 mg GAE/d DW and 147.41 mg GAE/g DW, respectively, for mature hulls). The same varieties also showed the highest values of RSA for green and mature hulls. 

The TPC values were higher than those reported by Siriwardhana et al. [25], who reported lower amounts of total phenolics (71.1 mg GAE/g DW) in an AH extract with 80% ethanol. In addition, TPC concentrations reported by Pinelo et al. [26] ranged from 23 to 61 mg GAE/g DW, which were lower than those obtained in the current study. The results obtained in this study were similar to those reported previously by Kahlaoui et al. [7], with TPC ranging from 133.6 to 210.4 mg GAE/g DW for seven AH varieties. These differences could be related to modifications in the extraction methods used or differences in the crop conditions and maturity of the fruits. 

### 3.2. Physico-Chemical Bread Characteristics 

Table 5 reports the physical properties (crumb colour, specific volume, and water activity) evaluated on breads. The colour is an important baking characteristic, in addition to texture and aroma, due to its contribution to consumer preference [27]. The colour analysis results indicate that the bread containing AH powder had a significantly darker (lower L* values) crumb than wheat bran and control breads. The bread crumb was darker in all breads containing AH powders, especially the one prepared with 8% hull powders, than in the wheat bran and control breads. Furthermore, the bread supplemented with 4% AH powder was significantly lighter in colour than those with 8% AH powder. In addition, the parameters a* (redness) and b* (yellowness) increased with increasing amounts of AH and wheat bran in all bread samples from 4 to 8%, indicating that the colour of the bread became increasingly reddish-black with the increase in bread yellowness.

For all formulations, breads substituted with hull powder had significantly higher b* values than breads with wheat bran flour and the control bread. This result may be due to the original yellow pigment present in AH and wheat bran flour. In particular, the crumb of the breads with higher percentages of AH changed colour from white to brown (lower lightness values). According to Sabanis et al. [28], the crumb colour is not affected by temperature, but may be influenced by the colour of the substituted flour because the crumb does not reach the high temperature of the crust.

The specific volume is used by consumers to evaluate the quality of fresh bread, considering that the increased loaf volume is the dominant factor in improved sensory quality of bread [29]. As shown in Table 5, the specific volume increased as the supplementation level of AH increased. However, the incorporation of this ingredient significantly decreased the volume of the breads compared to that of the control (2.58 cm^3^/g) and wheat bran bread (2.50 cm^3^/g), except for the bread produced with Achaak and Fakhfekh green powder, whose specific volumes were 2.84 and 2.64 cm^3^/g, respectively, with 8% added AH powder. This effect may be counteracted by increasing the water content of the dough and the water-holding capacity of the flours, which can increase the dough viscosity [30]. 

The specific volume of bread was significantly reduced (*p* < 0.05) with the addition of powders. These results are in good agreement with those reported by Della Gatta and Piergiovanni [31], who found that increased levels of substitution of sunflower meal in wheat flour bread yield loaves smaller in volume and lower specific volume. Additionally, Ragaee et al. [32] and Hathorn et al. [33] reported that partial substitution of wheat flour with barley, oat, rye, cellulose, and sweet-potato flour result in a reduction in the loaf specific volume of breads.

The water activity (a_w_) for all bread formulations ranged from 0.89 to 0.97. In this study, the replacement of refined wheat flour with AH powder did not significantly affect the a_w_ compared to that of the wheat bran and control breads. Furthermore, the a_w_ of the bread samples decreased with increased levels of AH. These results could be attributed to the fact that the water-holding capacity of wheat flour (0.79) is lower than that of hull powders and bran flour because of the lower fibre content. Our results are in agreement with those reported by Mau et al. [34], who found that the a_w_ of bread samples decreases with increased amounts of aerial parts of sweet-potato powders added.

Hardness is commonly used as an index of bread quality. All the breads obtained after substituting wheat flour with almond hull showed significantly (*p* < 0.05) lower hardness than the control bread obtained with only wheat flour (Table 6). The hardness of fresh bread was in the following order: control bread > wheat bran bread (8%) > wheat bran bread (4%) > AH bread (8%) > AH bread (4%), indicating that bread became harder as the AH powder concentration increased in the blend formulae. An increase in hardness might be attributed to the density of the tested bread, which is inversely correlated with its specific volume. These results disagree with those of Mau et al. [34], who found that bread supplemented with sweet-potato powder was significantly harder than the wheat flour bread and control. The hardness showed lesser differences between the almond varieties and between the hull origins, although flours obtained using green hulls have a significantly lower hardness than those obtained using mature hulls. This may be related to the lower fibre content of the green hulls compared to the mature hulls, and although hardness generally increased with the fibre content in AH powders, there were some exceptions [35]. Gumminess and chewiness of all bread formulations (Table 6) were positively correlated with hardness (r = 0.93 and 0.89, respectively). The AHs obtained from mature almonds were characterised by the highest insoluble fibre content, and the breads obtained showed the highest gumminess, chewiness, and lower resilience.

The gumminess and chewiness of breads increased with increased amounts of AH powder, whereas the addition of 8% wheat bran flour to the bread formulation significantly decreased (*p* < 0.05) the gumminess and chewiness of breads. The cohesiveness of all breads decreases when the amounts of AH powder and wheat bran flour increase; however, bread with high cohesiveness formed a bolus rather than disintegrating during mastication [36]. The springiness and resilience of breads followed the following order: AH (4%) > AH (8%) > wheat bran bread (4%) > wheat bran bread (8%) > control bread. 

Subsequently, substituting up to 4% of wheat flour with AH powder did not seem to cause changes in the textural properties of the bread, and the effects of the substitution on these properties were more noticeable at 8%.

According to the results of the image analysis, the crumb structure of bread prepared with AH powder had a larger gas-cell distribution than that of the control (Table 7). The wheat bran bread yielded a crumb matrix similar to that of the control, with an even distribution of air cells.

The introduction of fibre as wheat bran or AH powder resulted in an increase in the porosity of the bread loaf. However, increasing the addition decreased the percentage of the area occupied by the cell, especially when the mature hull was used. The latter phenomenon is determined by the higher content of insoluble fibre in the hull, which decreases the swelling capacity of the protein/fibre network. The dimensions of the pores (mean cell area) were not affected by the addition of bran or AH when added to the 4%, whereas a decrease in their dimensions was observed with a higher introduction, due to the fibre content of the matrix used, which determined a lower content of gluten network able to react to the expansion of the gases formed during the fermentation and baking processes.

Regarding the distribution of the different porous ranges, the most abundant cells were those with a dimension comprised between 0.1 and 0.4 mm^2^, representing approximately 40% of the total, followed by those with a dimension comprised between 0.4 and 1.6 mm^2^, representing 30% of the total (Table 8). In general, statistically significant differences were observed when the use of a mature hull resulted in a higher percentage of cells in the mentioned ranges than that of the green stage. Statistically significant differences were observed among the almond varieties for all the ranges and AH percentages.

The bread obtained with the addition of AH powders, particularly with green AHs, showed the highest phenolic content and antioxidant activity (Table 9) according to the high content of total phenolics in these ingredients, as reported in Table 4. In particular, the TPC of the functional bread was 7-fold higher and the RSA was 25-fold higher than that of wheat bread. For the green powder, the TPC and RSA values were two-fold higher at 8% of addition than that of 4% of addition. With the mature hull, the difference was about 1.5-fold. The breads produced with the Achaak green and mature powders showed the highest TPC and RSA values.

### 3.3. Sensory Evaluation

The sensory effect of AH powder addition on bread was evaluated using a consumer liking test (Table 10). 

According to the Kruskal–Wallis H-test results, the addition of 4% AH powder and wheat bran flour to the formulations did not affect the taste perceptions during consumption, although the addition of 8% bran flour resulted in increased levels of consumer acceptance compared to those of 8% almond hull breads. Generally, breads supplemented with 8% AH powder were more appreciated than those supplemented with 4% AH powder.

Similar results were obtained for flavour, where the consumer preference was influenced by type, maturity, and the level of substitution of flour, and breads supplemented with 8% of AH powder were the most appreciated. In contrast, the addition of cereal products, fruit by-products, or plant material adversely influences the aroma and overall preference of baked foods [37]. Tańska et al. [38] reported that the addition of fruit pomace (20%) from blackcurrant fruit, rowan, rosehip, and elderberry decreases the aroma score of shortbread cookies. Hayta et al. [39] showed a reduction in the overall preference of bread with the addition of 10% grape pomace powder. In addition, adding 5% ground green coffee bean powder significantly decreases bread aroma [40].

The overall liking reflects the consumer acceptance, and the breads obtained using AH green powders were the most liked at the 4% level, whereas the breads obtained using AH mature powders were preferred at the 8% level. This could be related to the high quantity of polyphenols with unpleasant, bitter, and astringent taste in the AH green powder. 

### 3.4. In Vitro Simulated GID

The in vitro simulated GID was performed for breads prepared with wheat bran, only with wheat flour, and containing 8% Achaak hulls that showed higher TPC and RSA values, as shown in Section 3.2 (Table 9). The results of the in vitro simulated GID are presented in Table 11. The TPC of the breads before simulated gastrointestinal digestion ranged from 52.95 to 450.69 mg GAE/100 g DW. The lowest content was found in wheat bran bread and bread produced only with wheat flour, whereas bread containing Achaak green hull showed the highest values.

After the in vitro GID, the bio-accessibility of the phenolic compounds depended on the samples, and therefore, the TPC decrease was higher for the bread containing wheat bran (23.71 mg GAE/100 g DW) and refined flour (20.91 mg GAE/100 g DW), with a decrease of 60% and 65%, respectively. In contrast, the bread containing green AH (272.88 mg GAE/100 g DW) and mature AH (237.24 mg GAE/100 g DW) showed a TPC reduction of 39.5% and 19.5%, respectively. Consequently, the bio-accessibility of the bio-active compounds was higher than 50%. Szawara et al. [41] reported that the polyphenol content increased by 8- and 11-fold in breads enriched with 50% each of white and roasted buckwheat groats, respectively. Similar results were obtained by Hidalgo et al. [42], who conducted an in vitro digestion of biscuits prepared from einkorn flour mixed with different proportions of amaranth, quinoa, and buckwheat. The RSA values before the digestion process ranged from 30.7 to 2204.5 μmol TE/100 g DW. As for TPC, the extract from bread containing 8% Achaak (green and mature) showed higher antioxidant capacity than those from wheat bran and control bread.

Generally, a reduction in the antioxidant capacity of up to 48% was observed in bread samples after GID, with values ranging from 34.32 to 1145.32 μmol TE/100 g DW. The control bread had lower losses of antioxidant activity (12%), whereas bread substituted with AH powders showed the highest losses after digestion (48%) compared with wheat bran bread, which had a reduction of 40%. However, the initial RSA values of the control and wheat breads were significantly lower than those observed for bread enriched with AH. Similar results have been reported by other authors. In particular, Hidalgo et al. [42] comparatively evaluated the antioxidant capacities of in vitro digested buckwheat and whole wheat flour enriched with quinoa and amaranth. Nevertheless, the digestion rates of bread with anthocyanin-rich black rice extract powder were reduced by 12.8%, 14.1%, and 20.5% for bread fortified with 1%, 2%, and 4% of extract powder, respectively [43].

## 4. Conclusions

In this study, a new bread formulation enriched at two concentrations (4% and 8%) with AH powders obtained from different almond cultivars was developed and evaluated. The results showed that the incorporation of AH powders significantly improved the fibre, ash, TPC, RSA, and fat content of the bread. Regarding the physical characteristics, breads supplemented with AH powders showed slightly lower specific volume, darker crumb colour, and lower gumminess, hardness, and chewiness, but similar cohesiveness, relative to the control. The bread containing 8% AH powder showed the highest scores for overall liking. Moreover, the in vitro bio-accessibility of polyphenols exceeded 60%, highlighting their potential to be absorbed in the gastrointestinal tract or have beneficial effects at the intestinal level. Finally, the information obtained in this study indicated that AH powder can replace 8% of wheat flour and might provide significant health improvement, and the resulting bread maybe recognised as a functional bread, reflecting several potential benefits. This application may facilitate the establishment of a circular economy around the almond industry by the re-introduction of by-products into the productive system. Further studies should be performed to evaluate the effects of this addition in vivo but also to define the effects of AH addition on other foods such as bakery products, fermented milks, and so on.

## Figures and Tables

**Table 1:** Ingredients (g) used for the production of control and functionalised breads with AHs and wheat bran.

	Control Bread	Bread with Wheat Bran		Bread with AHs	
		4% (*w*/*w*)	8% (*w*/*w*)	4% (*w*/*w*)	8% (*w*/*w*)
Wheat flour	520	499.2	478.4	499.2	478.4
AH powder				20.8	41.6
Wheat bran		20.8	41.6		
Salt	8.5	8.5	8.5	8.5	8.5
Sucrose	2.5	2.5	2.5	2.5	2.5
Yeast	3	3	3	3	3
Water	350	350	350	350	350

**Table 2:** Chemical composition of green and mature AHs and results of variance analysis with Duncan’s test (*p* < 0.05) performed between the almond varieties and the two harvesting times (green and mature).

	Moisture (%)		Ash (%)		Lipids (%)	
	Green	Mature	Green	Mature	Green	Mature
Achaak	8.15 ± 0.10 bA	9.80 ± 0.14 cB	8.18 ± 0.86 c	8.17 ± 0.84 b	1.29 ± 0.13 abA	2.50 ± 0.25 bB
Fakhfekh	7.11 ± 0.07 aB	6.01 ± 0.08 aA	7.44 ± 0.09 bA	12.07 ± 1.02 cB	1.47 ± 0.15 bA	2.45 ± 0.25 abB
Fascionello	8.16 ± 0.12 c	8.61 ± 0.12 b	6.98 ± 0.39 aA	8.37 ± 0.98 bB	2.55 ± 0.25 c	2.57 ± 0.25 b
Laurane	6.95 ± 0.11 bA	8.77 ± 0.12 bB	8.13 ± 0.41 c	8.75 ± 0.63 b	1.15 ± 0.12 aA	2.58 ± 0.25 bB
Pizzuta	8.75 ± 0.08 dB	6.80 ± 0.09 aA	7.98 ± 0.55 b	7.46 ± 0.61 a	2.39 ± 0.25 c	2.51 ± 0.24 a
Romana	9.01 ± 0.09 c	8.97 ± 0.12 b	10.45 ± 1.12 dB	8.50 ± 0.12 bA	2.65 ± 0.27 d	2.71 ± 0.27 c
Significance	***	**	***	**	**	*
	**Proteins (%)**		**Total Carbohydrates (%)**		**Glucose (mg/g)**	
	**Green**	**Mature**	**Green**	**Mature**	**Green**	**Mature**
Achaak	2.75 ± 0.28 aA	3.44 ± 0.44 bB	41.50 ± 0.51 bA	53.50 ± 0.12 cB	69.87 ± 0.32 cA	148.11 ± 2.78 eB
Fakhfekh	3.13 ± 0.03 ab	3.03 ± 0.06 ab	41.11 ± 0.38 bA	56.28 ± 0.34 eB	73.39 ± 0.12 cB	31.48 ± 4.35 aA
Fascionello	3.63 ± 0.48 b	3.44 ± 0.15 b	41.26 ± 0.01 bA	44.24 ± 0.18 abB	68.89 ± 0.72 cA	111.48 ± 1.06 bB
Laurane	2.88 ± 0.01 aA	3.75 ± 0.74 cB	40.55 ± 0.20 aA	55.77 ± 0.15 dB	77.23 ± 0.93 cA	122.53 ± 9.50 cB
Pizzuta	4.69 ± 0.11 cB	2.81 ± 0.37 aA	42.85 ± 0.76 bA	45.30 ± 0.56 bB	48.56 ± 0.63 bA	133.48 ± 3.91 dB
Romana	3.50 ± 0.16 b	3.06 ± 0.23 ab	41.08 ± 0.78 b	41.72 ± 0.22 a	19.28 ± 0.30 aA	103.47 ± 0.80 bB
Significance	**	**	**	***	***	***
	**Fructose (mg/g)**		**Xylose (mg/g)**		**Sorbitol (mg/g)**	
	**Green**	**Mature**	**Green**	**Mature**	**Green**	**Mature**
Achaak	45.12 ± 1.66 dA	56.27 ± 1.01 cB	1.88 ± 0.02 aA	4.41 ± 0.04 bB	21.06 ± 1.06 eA	46.03 ± 1.60 dB
Fakhfekh	23.09 ± 0.38 c	21.33 ± 2.81 a	2.02 ± 0.23 bA	4.42 ± 0.65 bB	7.29 ± 1.53 aA	15.01 ± 2.40 aB
Fascionello	42.93 ± 0.58 dA	61.16 ± 1.06 dB	5.60 ± 0.03 dA	6.66 ± 0.08 dB	18.30 ± 0.17 dA	21.75 ± 0.74 bB
Laurane	15.82 ± 1.13 bA	55.49 ± 4.28 cB	3.44 ± 0.02 cA	5.92 ± 0.36 cB	9.77 ± 0.97 bA	16.93 ± 0.80 aB
Pizzuta	6.67 ± 0.28 aA	37.07 ± 1.16 bB	2.58 ± 0.25 bA	3.20 ± 0.10 aB	16.29 ± 0.62 cdA	30.36 ± 0.62 cB
Romana	11.57 ± 0.25 aA	52.85 ± 1.02 cB	2.68 ± 0.17 bA	5.70 ± 0.11 cB	12.79 ± 0.27 cA	28.5 ± 0.84 bcB
Significance	***	***	**	**	**	***
	**Total Fibre (%)**		**Insoluble Fibre (%)**		**Soluble Fibre (%)**	
	**Green**	**Mature**	**Green**	**Mature**	**Green**	**Mature**
Achaak	26.66 ± 0.64 bA	34.59 ± 0.62 bB	20.54 ± 0.18 bcA	33.24 ± 0.82 bB	6.12 ± 0.20 eB	1.35 ± 0.05 aA
Fakhfekh	23.32 ± 0.24 aA	35.23 ± 0.59 cB	18.47 ± 0.39 aA	33.47 ± 0.21 bB	4.85 ± 0.19 cB	1.76 ± 0.17 bA
Fascionello	34.93 ± 0.82 c	35.77 ± 0.77 c	33.04 ± 0.17 d	33.70 ± 0.66 b	1.89 ± 0.18 aA	2.07 ± 0.11 dB
Laurane	25.15 ± 0.50 bA	27.65 ± 0.02 aB	19.31 ± 0.45 b	24.89 ± 0.32 a	5.84 ± 0.29 dB	1.76 ± 0.13 bA
Pizzuta	26.81 ± 0.23 b	25.32 ± 0.25 a	23.56 ± 0.21 c	22.32 ± 0.35 a	2.68 ± 0.25 b	2.05 ± 0.23 d
Romana	29.12 ± 0.13 bc	31.03 ± 0.10 a	26.03 ± 0.21 c	28.45 ± 0.74 ab	1.91 ± 0.12 a	1.90 ± 0.26 c
Significance	***	***	***	***	***	**
	**Malic Acid (mg/g)**		**Tartaric Acid (mg/g)**		**Citric Acid (mg/g)**	
	**Green**	**Mature**	**Green**	**Mature**	**Green**	**Mature**
Achaak	79.38 ± 7.90 dB	48.95 ± 0.41 dA	92.1 ± 0.22 bB	59.9 ± 0.14 cA	2.4 ± 0.01 a	2.85 ± 0.04 a
Fakhfekh	52.2 ± 17.05 dB	40.01 ± 0.05 cdA	104.9 ± 0.26 cB	55.5 ± 0.22 cA	6.65 ± 0.01 cB	5.95 ± 0.02 cA
Fascionello	18.20 ± 2.19 a	19.23 ± 0.39 a	55.25 ± 2.51 aB	36.85 ± 0.04 aA	5.85 ± 0.02 b	5.65 ± 0.23 c
Laurane	29.65 ± 0.03 bB	16.61 ± 1.18 aA	59.45 ± 0.06 aB	34.2 ± 0.08 aA	15.85 ± 0.10 e	11.55 ± 0.1 dA
Pizzuta	28.52 ± 3.15 bB	24.92 ± 3.23 bA	128.35 ± 2.01 dB	83.01 ± 0.33 dA	8.35 ± 0.02 dB	5.85 ± 0.14 cA
Romana	40.64 ± 4.12 cB	35.65 ± 3.96 cA	56.5 ± 0.45 aB	50.05 ± 2.41 bA	5.15 ± 0.02 bB	3.3 ± 0.02 bA
Significance	***	***	**	***	***	***

Data (mean ± standard deviation; *n* = 3) were expressed as dry weight (DW). Means followed by different lowercase letters indicate significant difference at *p* < 0.05 among almond varieties; means followed by different uppercase letters indicate significant difference at *p* < 0.05 between AH harvesting times; * *p* < 0.05, ** *p* < 0.01, *** *p* < 0.001.

**Table 3:** Values of water-holding capacity (WHC), oil-binding capacity (OBC), and swelling capacity (SWC) of AH powders, wheat bran, and wheat flour, and results of variance analysis with Duncan’s test (*p* < 0.05) performed between the products and the two harvesting times (green and mature).

	WHC (g water/g DW)		OBC (g oil/g DW)		SWC (mL/g DW)	
	Green	Mature	Green	Mature	Green	Mature
Achaak	4.52 ± 0.02 c	4. 30 ± 0.05 e	2.58 ± 0.26 d	2.35 ± 0.55 bc	4.75 ± 0.21 bcA	6.73 ± 0.77 dB
Fakhfekh	5.17 ± 0.06 dB	4.13 ± 0.29 eA	2.43 ± 0.13 cdB	2.17 ± 0.04 bA	4.86 ± 0.34 bcA	5.47 ± 0.15 cB
Fascionello	5.20 ± 0.12 dB	4.29 ± 0.34 eA	2.25 ± 0.07 bc	2.22 ± 0.08 bc	4.66 ± 0.08 bA	7.83 ± 0.72 e
Laurane	4.67 ± 0.42 cB	3.99 ± 0.30 deA	2.53 ± 0.08 d	2.43 ± 0.14 c	5.96 ± 0.27 dB	5.19 ± 0.27 bcA
Pizzuta	4.12 ± 0.26 cB	3.75 ± 0.02 cA	2.22 ± 0.01 bcB	2.04 ± 0.06 abA	5.10 ± 0.01 cB	4.53 ± 0.53 bA
Romana	4.55 ± 0.03 cB	3.95 ± 0.08 dA	2.16 ± 0.02 bB	1.99 ± 0.07 aA	6.58 ± 0.31 eB	4.59 ± 0.17 bA
Wheat bran	2.41 ± 0.40 b		2.28 ± 0.06 bc		4.57 ± 0.61 b	
Wheat flour	0.79 ± 0.03 a		1.99 ± 0.18 a		3.47 ± 0.19 a	
Significance	***	***	***	***	***	***

Data (mean ± standard deviation; *n* = 3) were expressed as dry weight (DW). Means followed by different lowercase letters indicate significant difference at *p* < 0.05 among powder; means followed by different uppercase letters indicate significant difference at *p* < 0.05 between AH harvesting times; *** *p* < 0.001.

**Table 4:** Total phenolic content (TPC; mg GAE/g DW) and radical-scavenging activity (RSA; µmol eq. Trolox/g DW) of AH powders, wheat bran, and wheat flour used for bread production, and results of variance analysis with Duncan’s test (*p* < 0.05) performed between the products and the two harvesting times (green and mature).

	TPC		RSA	
	Green	Mature	Green	Mature
Achaak	117.34 ± 2.04 bB	105.34 ± 3.99 bA	760.59 ± 35,10 cB	709.29 ± 7.03 cA
Fakhfekh	184.53 ± 7.09 eB	147.71 ± 4.82 dA	1045.72 ± 20.89 fB	915.94 ± 8.61 dA
Fascionello	159.57 ± 10.02 eB	115.20 ± 2.81 cA	934.21 ± 80,91 deB	704.31 ± 13.91 cA
Laurane	124.05 ± 1.37 cB	103.44 ± 1.37 cA	869.76 ± 3.27 cB	744.85 ± 3.27 cA
Pizzuta	160.55 ± 6.13 f	153.11 ± 2.74 d	1159.83 ± 27.84 fB	881.50 ± 12.88 eA
Romana	149.19 ± 4.29 dB	113.02 ± 3.73 cA	962.88 ± 29.00 eB	671.78 ± 10.76 bA
Wheat bran	3.82 ± 0.09 a		39.49 ± 0.13 b	
Wheat flour	1.13 ± 0.03 a		0.45 ± 0.02 a	
Significance	***	***	***	***

Data (mean ± standard deviation; *n* = 3) were expressed as dry weight (DW). Means followed by different lowercase letters indicate significant difference at *p* < 0.05 among products; means followed by different uppercase letters indicate significant difference at *p* < 0.05 between AH harvesting times; *** *p* < 0.001.

**Table 5:** Values of crumb colour, specific volume, and water activity evaluated on the control breads and breads with 4% and 8% of AH powders and wheat bran, and results of variance analysis with Duncan’s test (*p* < 0.05) performed between the products and the two harvesting times (green and mature).

Powder Addition		Redness (a*)		Yellowness (b*)		Lightness (L*)		Specific Volume (VS, cm^3^/g)		Water Activity (aw, 25 °C)	
		Green	Mature	Green	Mature	Green	Mature	Green	Mature	Green	Mature
4%	Achaak	5.66 ± 0.16 dA	6.09 ± 0.51 dB	18.14 ± 0.88 cB	15.90 ± 1.70 cA	45.76 ± 1.94 c	42.49 ± 4.55 b	1.86 ± 0.04 bA	2.14 ± 0.02 bcB	0.96 ± 0.008 b	0.96 ± 0.004 b
	Fakhfekh	4.85 ± 0.1 c	5.11 ± 0.24 c	17.94 ± 0.05 cB	14.85 ± 0.54 abcA	48.74 ± 0.52 dB	40.70 ± 2.34 abA	2.46 ± 0.10 dB	2.04 ± 0.02 bA	0.94 ± 0.003 a	0.93 ± 0.168 a
	Fascionello	8.38 ± 0.18 fB	7.61 ± 0.47 dA	18.46 ± 0.82 cB	14.97 ± 1.03 abcA	39.21 ± 1.96 aA	38.23 ± 1.13 ab	2.08 ± 0.02 cB	2.01 ± 0.001 aA	0.97 ± 0.006 b	0.96 ± 0.004 b
	Laurane	6.67 ± 1.01 e	5.72 ± 0.08 d	20.28 ± 1.41 dB	14.51 ± 0.83 abcA	47.00 ± 0.82 cdB	37.75 ± 3.77 abA	1.72 ± 0.06 aA	2.22 ± 0.16 cB	0.97 ± 0.002 b	0.97 ± 0.006 b
	Pizzuta	7.32 ± 0.27 e	7.24 ± 0.28 d	19.63 ± 0.66 dB	15.16 ± 0.46 bcA	42.34 ± 0.96 bB	41.01 ± 0.58 abA	2.12 ± 0.02 c	2.15 ± 0.03 bc	0.97 ± 0.015 b	0.96 ± 0.004 b
	Romana	4.67 ± 0.13 cA	7.18 ± 0.17 dB	15.09 ± 0.41 b	14.73 ± 0.18 abc	40.37 ± 1.68 abA	39.76 ± 0.53 ab	2.20 ± 0.02 c	2.16 ± 0.02 bc	0.97 ± 0.001 b	0.96 ± 0.007 b
	Wheat bran	0.90 ± 0.39 b		13.21 ± 1.57 a		57.60 ± 1.05 e		2.50 ± 0.04 d		0.97 ± 0.009 b	
	Wheat flour	−0.31 ± 0.46 a		13.42 ± 0.44 ab		60.66 ± 0.20 f		2.58 ± 0.04 d		0.97 ± 0.006 ab	
Significance		***	***	***	ns	***	***	***	***	**	**
8%	Achaak	7.58 ± 1.13 d	9.11 ± 0.45 e	20.14 ± 2.14 bc	18.05 ± 0.64 d	43.47 ± 0.70 cB	38.11 ± 1.01 dA	2.84 ± 0.23 dB	2.48 ± 0.04 bcA	0.96 ± 0.005 b	0.89 ± 0.086 a
	Fakhfekh	6.07 ± 0.28 cA	6.84 ± 0.08 cB	19.61± 0.58 bcB	15.52 ± 0.37 cA	47.87 ± 0.52 eB	36.53 ± 1.62 bA	2.60 ± 0.13 cdB	2.10 ± 0.02 aA	0.93 ± 0.019 a	0.90 ± 0.001 ab
	Fascionello	10.42 ± 0.25 f	10.21 ± 0.32 g	20.85± 0.55 bcB	17.38 ± 0.63 dA	37.44 ± 1.27 a	36.35 ± 0.11 b	2.23 ± 0.03 a	2.17 ± 0.03 b	0.96 ± 0.004 b	0.89 ± 0.003 a
	Laurane	7.75 ± 0.07 dB	7.50 ± 0.13 dA	21.69 ± 0.22 cB	15.42 ± 0.47 cA	45.84 ± 0.45 dB	35.28 ± 1.58 aA	2.47 ± 0.004 bc	2.52 ± 0.16 bc	0.94 ± 0.009 b	0.93 ± 0.001 b
	Pizzuta	9.37 ± 0.14 eA	9.81 ± 0.02 fgB	20.09 ± 0.73 bcB	17.17 ± 0.13 dA	36.58 ± 1.17 aA	38.66 ± 0.20 eB	2.17 ± 0.06 a	2.32 ± 0.03 bc	0.96 ± 0.002 b	0.93 ± 0.001 b
	Romana	7.15 ± 0.46 dA	9.42 ± 0.24 efB	19.14 ± 0.51 bcB	15.82 ± 0.66 cA	40.79 ± 1.68 bB	34.80 ± 1.13 aA	2.29 ± 0.08 ab	2.25 ± 0.04 b	0.96 ± 0.003 b	0.89 ± 0.003 a
	Wheat bran	1.51 ± 0.30 b		13.55 ± 0.95 a		51.90 ± 1.46 f		2.53 ± 0.10 bc		0.96 ± 0.001 b	
	Wheat flour	−0.31 ± 0.46 a		13.42 ± 0.44 a		60.66 ± 0.20 g		2.58 ± 0.04 c		0.97 ± 0.006 b	
Significance		***	***	***	***	***	***	***	**	**	**

Data (mean ± standard deviation; *n* = 6) followed by different lowercase letters indicate significant difference at *p* < 0.05 among bread samples; data followed by different uppercase letters indicate significant difference at *p* < 0.05 between AH harvesting times; *** *p* < 0.001; ** *p* < 0.01; ns not significant.

**Table 6:** Values of texture parameters evaluated on breads and results of variance analysis with Duncan’s test (*p* < 0.05) performed between the products and the two harvesting times (green and mature).

Powder Addition		Hardness (N)		Cohesiveness (-)		Gumminess (N)	
		Green	Mature	Green	Mature	Green	Mature
4%	Achaak	9.14 ± 2.579 aA	32.55 ± 20.194 aB	0.82 ± 0.015 dB	0.65 ± 0.004 abA	7.53 ± 11,628 aA	21.22 ± 0.676 aB
	Fakhfekh	11.32 ± 1.506 aA	32.63 ± 3.276 aB	0.81 ± 0.009 cdB	0.59 ± 0.005 aA	9.18 ± 4.636 aA	19.30 ± 0.535 aB
	Fascionello	28.35 ± 9.594 a	37.636 ± 11.473 a	0.59 ± 0.012 ab	0.60 ± 0.026 a	16.69 ± 5.427 a	25.29 ± 8.117 a
	Laurane	11.16 ± 1.588 aA	25.56 ± 4.567 aB	0.77 ± 0.017 c	0.73 ± 0.019 c	8.67 ± 0.801 aA	18.93 ± 2.732 aB
	Pizzuta	28.83 ± 9.617 a	32.82 ± 1.897 a	0.59 ± 0.038 ab,	0.67 ± 0.052 bc	17.05 ± 5.865 a	16.13 ± 1.651 a
	Romana	39.38 ± 15.787 a	28.01 ± 2.781 a	0.56 ± 0.010 aA	0.64 ± 0.030 abB	22.27 ± 8.482 a	17.93 ± 1.538 a
	Wheat bran	63.28 ± 62.75 ab		0.61 ± 0.01 b		58.13 ± 37.95 ab	
	Wheat flour	145.73 ± 98.698 e		0.59 ± 0.047 ab		89.69 ± 67.514 b	
Significance		***	***	***	*	***	***
8%	Achaak	72.72 ± 53.27 ab	51.91 ± 0.00 a	0.59 ± 0.04 bA	0.70 ± 0.01 dB	42.09 ± 28.21 ab	36.85 ± 0.06 ab
	Fakhfekh	52.79 ± 37.12 abB	50.25 ± 13.96 aA	0.56 ± 0.08 b	0.54 ± 0.01 a	28.24 ± 16.61 a	27.64 ± 8.15 a
	Fascionello	33.06 ± 10.52 a	41.88 ± 12.60 a	0.57 ± 0.02 b	0.56 ± 0.00 ab	19.08 ± 6.43 a	21.62 ± 7.51 a
	Laurane	30.53 ± 4.04 a	37.89 ± 1.21 a	0.63 ± 0.02 b	0.58 ± 0.00 abc	19.26 ± 2.76 a	22.12 ± 0.89 a
	Pizzuta	24.60 ± 5.55 a	32.04 ± 5.98 a	0.56 ± 0.03 b	0.61 ± 0.00 c	13.95 ± 3.79 a	19.84 ± 3.79 a
	Romana	60.47 ± 21.45 abB	29.23 ± 7.53 aA	0.48 ± 0.002 aA	0.59 ± 0.01 bcB	29.19 ± 8.81 a	17.37 ± 4.75 a
	Wheat bran	95.36 ± 11.733 a		0.62 ± 0.027 b		38.52 ± 6.196 a	
	Wheat flour	145.73 ± 98.698 e		0.59 ± 0.047 ab		89.69 ± 67.514 b	
Significance		***	***	***	*	***	**
**Powder** **Addition**		**Springiness (mm)**		**Chewiness (mJ)**		**Resilience (-)**	
		**Green**	**Mature**	**Green**	**Mature**	**Green**	**Mature**
4%	Achaak	12.88 ± 2.135 abA	14.45 ± 0.197 cB	81.53 ± 5.49 aA	305.61 ± 14.42 aB	0.51 ± 0.0963 dB	0.35 ± 0.0002 bA
	Fakhfekh	14.94 ± 1868 c	14.02 ± 0.245 bc	100.35 ± 3.35 aA	270.88 ± 7.99 aB	0.49 ± 0.1014 cdA	0.32 ± 0.0069 abB
	Fascionello	14.04 ± 0.283 bc	14.15 ± 0.222 c	235.34 ± 4.84 a	358.34 ± 11.19 a	0.30 ± 0.0066 bA	0.32 ± 0.0088 abB
	Laurane	11.32 ± 1.158 aA	14.55 ± 0.148 cB	99.48 ± 2.50 aA	275.82 ± 5.04 aB	0.46 ± 0.0249 cB	0.43 ± 0.0134 eA
	Pizzuta	13.68 ± 0.160 bA	14.06 ± 0.128 bcB	238.43 ± 8.68 a	231.86 ± 2.35 a	0.31 ± 0.4007 b	0.37 ± 0.0374 c
	Romana	12.55 ± 0.125 abA	14.29 ± 0.100 cB	280.39 ± 6.37 a	256.40 ± 2.68 a	0.28 ± 0.1148 abA	0.35 ± 0.0347 bB
	Wheat bran	11.02 ± 0.33 a		811.62 ± 41.33 ab		0.24 ± 0.02 a	
	Wheat flour	9.68 ± 0.416 a		1241.31 ± 96.68 b		0.22 ± 0.0243 a	
Significance		***	*	***	***	***	**
8%	Achaak	12.64 ± 6.51 bB	10.23 ± 1.06 abA	547.03 ± 40.12 abB	303.14 ± 23.79 aA	0.27 ± 0.06 b	0.30 ± 0.00 cde
	Fakhfekh	13.39 ± 0.61 bc	13.70 ± 0.07 bc	373.27 ± 20.52 a	378.95 ± 43.22 a	0.25 ± 0.00 ab	0.26 ± 0.08 ab
	Fascionello	13.78 ± 0.03 bc	13.64 ± 0.13 bc	263.311 ± 9.73 a	296.09 ± 10.54 a	0.28 ± 0.02 b	0.27 ± 0.00 abc
	Laurane	10.04 ± 4.76 abA	13.93 ± 0.04 bcB	159.64 ± 9.43 aA	308.30 ± 11.59 aB	0.31 ± 0.00 bA	0.28 ± 0.01 bcd
	Pizzuta	13.97 ± 0.39 bcA	14.37 ± 0.17 cB	191.88 ± 5.57 aA	279.47 ± 6.66 aB	0.28 ± 0.02 bA	0.32 ± 0.00 eB
	Romana	12.03 ± 0.52 aB	14.00 ± 0.06 cB	348.37 ± 8.69 a	243.51 ± 7.57 a	0.29 ± 0.03 bA	0.30 ± 0.01 deB
	Wheat bran	9.99 ± 0.228 a		539.34 ± 7.60 ab		0.23 ± 0.0405 a	
	Wheat flour	9.68 ± 0.416 a		1241.31 ± 96.68 b		0.22 ± 0.0243 a	
Significance		***	***	***	***	***	***

Data (mean ± standard deviation; *n* = 8) followed by different lowercase letters indicate significant difference at *p* < 0.05 among breads; data followed by different uppercase letters indicate significant difference at *p* < 0.05 between AH harvesting times; *** *p* < 0.001; ** *p* < 0.01; * *p* < 0.05.

**Table 7:** Results of image-analysis parameters evaluated on the breads substituted with AH powders and results of variance analysis with Duncan’s test (*p* < 0.05) performed between the products and the two harvesting times (green and mature).

Powder Addition		Mean Cell Area (mm^2^)		Cell Density (Cells/mm^2^)		Circularity		Percent of Area (%)	
		Green	Mature	Green	Mature	Green	Mature	Green	Mature
4%	Achaak	2.02 ± 0.19 bA	2.74 ± 1.76 bB	215.94 ± 37.42 cA	275.70 ± 49.15 cB	0.29 ± 0.009 abcB	0.27 ± 0.02 aA	34.81 ± 1.04 dB	32.60 ± 1.82 bcA
	Fakhfekh	1.86 ± 0.28 a	1.76 ± 0.23 a	221.22 ± 53.68 cA	270.80 ± 55.09 cB	0.31 ± 0.01 cB	0.29 ± 0.013 bA	29.49 ± 1.59 abA	33.04 ± 1.99 cB
	Laurane	2.03 ± 0.17 bA	1.72 ± 0.04 aB	201.78 ± 34.04 cA	277.25 ± 8.02 cB	0.31 ± 0.01 cB	0.28 ± 0.01 abA	32.66 ± 1.79 cdA	33.48 ± 1.48 cB
	Pizzuta	1.90 ± 0.07 aB	1.54 ± 0.83 aA	259.73 ± 27.50 dA	344.06 ± 10.77 dB	0.30 ± 0.05 bc	0.29 ± 0.001 b	33.14 ± 0.98 f	33.11 ± 1.54 c
	Romana	2.89 ± 0.69 bA	1.67 ± 0.01 aB	220.12 ± 37.83 cA	264.34 ± 8.07 cB	0.29 ± 0.02 abcB	0.27 ± 0.01 aA	28.88 ± 0.64 abA	30.30 ± 0.39 bB
	Fascionello	1.83 ± 0.22 aA	2.08 ± 0.12 bB	145.57 ± 38.14 aA	193.60 ± 19.22 bB	0.26 ± 0.09 a	0.27 ± 0.004 ab	30.66 ± 0.41 bA	33.46 ± 2.18 dB
	Wheat bran	2.17 ± 0.19 b		169.78 ± 19.80 ab		0.28 ± 0.03 ab		31.85 ± 1.99 abB	
	Wheat flour	1.61 ± 0.27 a		236.51 ± 33.05 cd		0.30 ± 0.02 bc		26.77 ± 2.38 a	
Significance		ns	ns	***	***	***	ns	***	***
8%	Achaak	1.37 ± 0.07 aA	1.61 ± 0.01 abB	361.61 ± 4.03 bcB	316.83 ± 6.83 cA	0.35 ± 0.01 dB	0.26 ± 0.02 aA	28.01 ± 2.16 abB	20.63 ± 0.73 aA
	Fakhfekh	1.73 ± 0.46 bA	1.60.91 ± 0.08 abB	273.64 ± 14.70 cA	293.69 ± 36.92 bcB	0.28±0.009 bcA	0.30 ± 0.002 bcB	30.29 ± 3.44 bc	30.06 ± 7.26 b
	Laurane	1.31 ± 0.07 aA	1.82 ± 0.12 bcB	383.35 ± 4.17 cB	233.04 ± 42.30 abA	0.32 ± 0.004 cdB	0.29 ± 0.02 bA	26.40 ± 1.19 aA	30.54 ± 0.82 bB
	Pizzuta	1.58 ± 0.10 ab	1.60 ± 0.11 ab	289.72 ± 3.87 bcA	306.21 ± 40.83 bcB	0.27 ± 0.004 aA	0.31 ± 0.01 cB	29.01 ± 0.54 abA	31.12 ± 0.24 cdB
	Romana	1.85 ± 0.11 bB	1.42 ± 0.03 aA	239.06 ± 44.15 bA	340.38 ± 2.85 cB	0.28 ± 0.004 bcB	0.29 ± 0.01 bA	32.39 ± 0.25 cdB	28.98 ± 0.71 abA
	Fascionello	2.16 ± 0.14 cB	1.70 ± 0.22 bA	185.84 ± 39.56 aA	295.81 ± 11.15 bcB	0.28 ± 0.004 bcB	0.29 ± 0.01 bA	34.28 ± 0.59 cB	32.49 ± 0.17 dA
	Wheat bran	1.92 ± 0.11 c		221.72 ± 26.21 ab		0.30 ± 0.01 cd		32.78 ± 0.05 d	
	Wheat flour	1.61 ± 0.27 ab		236.51 ± 33.05 b		0.30 ± 0.02 b		26.77 ± 2.38 ab	
Significance		***	***	***	***	**	**	***	**

Data (mean ± standard deviation; *n* = 8) followed by different lowercase letters indicate significant difference at *p* < 0.05 among products; data followed by different uppercase letters indicate significant difference at *p* < 0.05 between AH harvesting times; *** *p* < 0.001; ** *p* < 0.01; ns not significant.

**Table 8:** Values of cell distribution for each cell area in the breads substituted with AH powders and results of variance analysis with Duncan’s test (*p* < 0.05) performed between the products and the two harvesting times (green and mature).

Powder Addition	Cell Area (mm^2^)	Cells Distribution (%)^a^									
			Achaak	Fakhfekh	Laurane	Pizzuta	Romana	Fascionello	Wheat Bran	Wheat Flour	Significance
4%	<0.1	Green	0.33 ± 0.08 c	0.24 ± 0.02 bc	0.12 ± 0.04 bA	0.23 ± 0.36 eA	0.16 ± 0.03 bB	0.09 ± 0.001 a	0.41 ± 0.08 d	0.34 ± 0.08 c	**
		Mature	0.22 ± 0.01 bc	0.1 ± 0.04 b	0.13 ± 0.02 dB	0.20 ± 0.05 cB	0.22 ± 0.01 aA	0.24 ± 0.05 a			**
	0.1–0.2	Green	26.06 ± 2.07 b	27.22 ± 1.79 b	25.90 ± 0.78 bA	25.15 ± 1.18 cB	28.12 ± 0.77 aA	43.70 ± 16.31 cB	25.75 ± 0.12 b	28.65 ± 0.64 b	***
		Mature	26.70 ± 2.10 a	25.01 ± 1.95 a	29.06 ± 1.25 cB	29.04 ± 3.46 aA	23.69 ± 1.78 cB	29.24 ± 4.62 bA			***
	0.2–0.4	Green	18.93 ± 1.89 abA	19.78 ± 1.78 bA	19.58 ± 1.30 bA	18.27 ± 2.53 cB	21.26 ± 0.44 cB	17.98 ± 1.92 aA	21.51 ± 0.96 c	23.22 ± 0.59 d	**
		Mature	20.45 ± 1.11 bB	20.61 ± 0.58 bB	21.63 ± 0.60 cB	19.37 ± 3.52 abA	18.81 ± 2.98 aA	22.39 ± 2.71 dB			**
	0.4–0.8	Green	16.76 ± 1.77 b	16.49 ± 1.64 b	17.11 ± 0.38 cB	16.75 ± 0.92 a	18.69 ± 0.21 cB	16.14 ± 3.75 bA	15.55 ± 1.19 ab	16.55 ± 1.05 b	**
		Mature	17.01 ± 0.90 d	17.78 ± 1.42 d	15.90 ± 0.53 aA	18.72 ± 2.18 c	19.16 ± 2.81 bA	16.95 ± 1.66 eB			***
	0.8–1.6	Green	12.52 ± 1.27 c	13.76 ± 1.97 c	14.48 ± 1.66 cB	17.07 ± 1.27 bA	13.07 ± 0.55 cB	5.57 ± 7.59 aA	14.43 ± 0.77 c	13.01 ± 1.69 c	***
		Mature	13.58 ± 2.01 c	14.84 ± 0.43 c	11.93 ± 1.08 bA	13.88 ± 0.80 cB	14.83 ± 0.19 bA	14.24 ± 3.79 bB			***
	1.6–3.2	Green	9.58 ± 0.49 c	9.44 ± 2.50 c	9.55 ± 1.28 cB	10.63 ± 0.66 aA	7.67 ± 1.36 dB	8.81 ± 3.01 bB	9.67 ± 0.32 c	9.07 ± 0.19 c	***
		Mature	9.37 ± 2.07 c	9.74 ± 0.50 c	9.63 ± 0.72 aA	9.12 ± 0.74 dB	11.85 ± 2.44 aA	10.16 ± 3.35 aA			***
	3.2–6.4	Green	8.13 ± 0.86 dB	7.23 ± 1.44 cd	6.24 ± 1.05 cB	7.19 ± 0.55 aA	5.49 ± 0.84 cB	4.17 ± 5.42 bB	5.98 ± 0.99 b	4.09 ± 1.29 b	***
		Mature	6.88 ± 0.27 cA	6.24 ± 0.60 c	5.25 ± 1.16 aA	5.53 ± 0.48 cB	6.31 ± 0.10 aA	7.06 ± 0.78 aA			***
	6.4–12.8	Green	4.57 ± 0.89 d	3.81 ± 0.71 c	4.51 ± 0.77 dB	2.42 ± 0.53 b	3.08 ± 0.39 bB	0.98 ± 1.70 aA	3.69 ± 1.06 c	3.02 ± 1.44 c	**
		Mature	3.82 ± 0.53 c	3.59 ± 0.34 c	4.25 ± 0.18 aA	2.42 ± 1.26 b	3.42 ± 0.18 aA	4.08 ± 0.89 bB			**
	12.8–25.6	Green	2.15 ± 0.32 cB	1.22 ± 0.48 b	1.30 ± 0.66 bB	1.10 ± 0.05 a	1.36 ± 0.15 bB	0.82 ± 1.42 a	1.78 ± 0.63 b	1.68 ± 0.74 b	**
		Mature	1.49 ± 0.39 cA	1.42 ± 0.15 c	1.55 ± 0.65 aA	1.10 ± 0.26 b	1.10 ± 0.08 aA	2.56 ± 0.67 b			***
	25.6–50	Green	0.87 ± 0.17 dB	0.38 ± 0.18 b	0.81 ± 0.49 dB	0.76 ± 0.15 abA	0.90 ± 0.07 cB	0.16 ± 0.28 a	0.82 ± 0.41 d	0.24 ± 0.06 ab	***
		Mature	0.22 ± 0.01 dA	0.40 ± 0.05 e	0.47 ± 0.11 aA	0.55 ± 0.32 eB	0.44 ± 0.03 bA	0.75 ± 0.04 c			***
			**Achaak**	**Fakhfekh**	**Laurane**	**Pizzuta**	**Romana**	**Fascionello**	**Wheat Bran**	**Wheat flour**	**Significance**
8%	<0.1	Green	0.11 ± 0.05 b	0.10 ± 0.04 b	0.17 ± 0.03 b	0.21 ± 0.001 aA	1.12 ± 0.04 cB	0.23 ± 0.02 a	0.36 ± 0.19 d	0.34 ± 0.08 d	**
		Mature	0.19 ± 0.08 b	0.07 ± 0.12 ab	0.11 ± 0.05 b	1.39 ± 0.06 cB	0.19 ± 0.02 aA	0.19 ± 0.04 a			**
	0.1–0.2	Green	25.79 ± 1.94 aA	28.09 ± 0.11 abA	29.07 ± 3.23 b	24.42 ± 0.18 dB	43.13 ± 20.20 cA	25.23 ± 2.92 cA	27.73 ± 0.99 ab	28.65 ± 0.64 ab	***
		Mature	40.78 ± 22.46 cB	27.64 ± 2.06 bB	29.00 ± 1.41 a	45.00 ± 4.07 cA	23.49 ± 0.55 dB	29.23 ± 16.20 dB			***
	0.2–0.4	Green	22.21 ± 0.74 b	25.27 ± 3.02 cB	24.04 ± 2.06 bB	21.81 ± 0.74 aA	23.16 ± 3.92 bA	18.94 ± 0.84 dB	21.24 ± 4.04 b	23.22 ± 0.59 b	***
		Mature	21.78 ± 2.19 b	23.00 ± 2.02 bA	21.18 ± 1.30 bA	20.28 ± 2.04 aB	20.77 ± 0.11 cB	19.96 ± 2.14 bcA			**
	0.4–08	Green	18.39 ± 0.70 dB	18.25 ± 1.31 dB	16.60 ± 1.07 c	18.31 ± 0.24 b	11.72 ± 2.72 bB	17.36 ± 0.77 aA	16.26 ± 1.97 c	16.55 ± 1.05 c	**
		Mature	11.31 ± 6.57 bcA	16.17 ± 1.39 bcA	17.34 ± 0.23 c	13.89 ± 1.64 bc	19.28 ± 2.08 aA	15.11 ± 0.10 bB			***
	0.8–1.6	Green	13.83 ± 1.58 dB	15.83 ± 3.05 eB	12.89 ± 1.27 d	13.86 ± 1.04 aA	7.46 ± 1.94 c	14.12 ± 1.98 b	12.64 ± 1.24 d	13.01 ± 1.69 d	***
		Mature	10.03 ± 9.47 cA	15.49 ± 2.38 dA	11.20 ± 0.25 c	3.33 ± 0.71 aB	15.66 ± 0.57 b	14.27 ± 0.27 a			***
	1.6–3.2	Green	10.22 ± 1.92 dB	10.07 ± 2.19 d	8.50 ± 1.46 bc	10.56 ± 1.48 a	5.60 ± 1.68 bB	10.08 ± 0.90 aA	9.15 ± 0.12 c	9.07 ± 0.19 c	***
		Mature	7.81 ± 1.61 cA	9.90 ± 2.82 c	8.50 ± 0.05 c	0.00 ± 0.00 a	10.62 ± 0.81 aA	10.29 ± 2.06 bB			***
	3.2–6.4	Green	5.92 ± 0.34 bcB	7.50 ± 1.43 dB	5.23 ± 1.23 bc	6.43 ± 0.28 aA	3.56 ± 0.03 bB	6.89 ± 0.83 a	6.47 ± 0.55 c	4.09 ± 1.29 b	***
		Mature	3.70 ± 5.23 bA	7.42 ± 1.20 dA	5.57 ± 0.46 d	4.72 ± 0.75 cB	5.90 ± 0.94 aA	5.60 ± 0.50 a			***
	6.4–12.8	Green	2.61 ± 0.12 c	3.47 ± 2.93 dB	2.07 ± 1.22 cA	2.60 ± 0.87 a	1.35 ± 0.09 bB	3.63 ± 0.46 cB	3.13 ± 2.51 d	3.02 ± 1.44 d	**
		Mature	2.04 ± 2089 c	2.65 ± 0.67 bA	4.07 ± 0.05 dB	2.12 ± 0.26 a	2.59 ± 0.54 aA	3.35 ± 0.65 aA			**
	12.8–25.6	Green	0.61 ± 0.32 b	1.28 ± 0.07 cB	1.17 ± 0.29 c	0.98 ± 0.48 aA	1.70 ± 0.22 cB	2.27 ± 1.19 a	1.75 ± 0.39 c	1.68 ± 0.74 c	**
		Mature	0.58 ± 0.83 b	0.70 ± 0.53 bA	2.29 ± 0.76 d	6.67 ± 0.43 eB	1.19 ± 0.63 aA	1.29 ± 0.05 a			**
	25.6–50	Green	0.10 ± 0.14 b	0.33 ± 0.68 c	0.14 ± 0.10 b	0.54 ± 0.14 a	0.29 ± 0.01 bc	0.86 ± 0.36 a	1.03 ± 0.77 d	0.24 ± 0.06 bc	**
		Mature	0.00 ± 0.00 a	0.27 ± 0.04 b	0.62 ± 0.07 c	0.00 ± 0.00 a	0.26 ± 0.12 a	0.71 ± 0.01 a			**

Data (mean ± standard deviation; *n* = 8) followed by different lowercase letters indicate significant difference at *p* < 0.05 among products; data followed by different uppercase letters indicate significant difference at *p* < 0.05 between AH harvesting times; *** *p* < 0.001; ** *p* < 0.01; ns not significant.

**Table 9:** Total phenolic content (TPC) and radical-scavenging activity (RSA) of breads fortified with AH powders, and results of variance analysis with Duncan’s test (*p* < 0.05) performed between the products and the two harvesting times (green and mature).

Powder Addition		TPC (mg GAE/100 g DW)		RSA (μmol TE/100 g DW)	
		Green	Mature	Green	Mature
4%	Achaak	237.93 ± 10.52 iB	170.17 ± 4.42 eA	1258.03 ± 57.89 fB	806.61 ± 46.10 eA
	Fakhfekh	197.56 ± 6.85 fB	116.63 ± 3.62 bA	860.35 ± 17.56 dB	592.20 ± 38.24 cA
	Fascionello	155.42 ± 3.94 cB	138.54 ± 4.14 cA	789.39 ± 30.92 dB	629.45 ± 23.97 cdA
	Laurane	166.73 ± 4.68 d	126.14 ± 3.07 dA	635.45 ± 28.93 cB	505.96 ± 39.12 cA
	Pizzuta	153.08 ± 4.83 cb	124.56 ± 3.55 bcA	657.11 ± 20.94 cB	536.45 ± 10.82 cA
	Romana	188.21 ± 2.54 eB	148.18 ± 5.20 dA	1040.86 ± 26.58 eB	726.60 ± 26.68 dA
	Wheat bran	59.91 ± 3.18 a		41.30 ± 4.04 b	
	Wheat flour	52.95 ± 1.74 a		30.69 ± 2.34 a	
	Significance	***	***	***	***
		**Green**	**Mature**	**Green**	**Mature**
8%	Achaak	450.69 ± 8.29 gB	294.74 ± 6.75 gA	2204.49 ± 119.37 iB	1498.20 ± 57.85 eA
	Fakhfekh	332.43 ± 4.78 fB	203.43 ± 6.74 fA	1607.47 ± 68.14 eB	814.94 ± 32.66 dA
	Fascionello	196.79 ± 3.40 cA	190.66 ± 12.56 dA	946.37 ± 30.49 cB	746.90 ± 30.39 cA
	Laurane	225.20 ± 4.61 dB	208.70 ± 7.79 cA	965.56 ± 23.62 cB	740.23 ± 32.48 cA
	Pizzuta	211.09 ± 11.19 dA	183.79 ± 0.71 cA	1050.31 ± 14.96 dB	876.98 ± 36.21 cdA
	Romana	319.94 ± 7.17 eB	223.99 ± 7.58 eA	1933.95 ± 83.74 fB	962.25 ± 20.90 dA
	Wheat bran	61.22 ± 1.05 b		87.75 ± 4.90 b	
	Wheat flour	52.95 ± 1.74 a		30.69 ± 2.34 a	
	Significance	***	***	***	***

Data (mean ± standard deviation; *n* = 6) were expressed as dry weight (DW). Data followed by different lowercase letters indicate significant difference at *p* < 0.05 among products; data followed by different uppercase letters indicate significant difference at *p* < 0.05 for different AH harvesting times; *** *p* < 0.001.

**Table 10:** Sum of the ranks for each parameter evaluated for the breads during the consumer tests and results of Kruskal–Wallis tests performed on the sum of the rank.

Powder Addition		Aspect		Colour		Taste		Flavour		Texture		Overall Liking	
		Green	Mature	Green	Mature	Green	Mature	Green	Mature	Green	Mature	Green	Mature
4%	Achaak	1555 bB	861 bA	1499 bB	938 abA	1348 bB	819 aA	1050 abB	805 abA	1276 bB	1049 aA	1096 abB	872 abA
	Fakhfekh	1266 abB	500 aA	1247 abB	587 abA	935 abB	454 aA	1000 abB	407 aA	1187 bB	384 aA	1080 abB	397 aA
	Fascionello	873 aA	1007 bB	920 ab	991 ab	1140 abB	752 aA	1281 bB	930 abA	1060 abB	889 aA	1302 bB	883 abA
	Laurane	825 aB	415 aA	748 aB	452 aA	746 aB	357 aA	664 aB	403 aA	465 aB	391 aA	663 aB	434 aA
	Pizzuta	917 ab	985 b	1031 abB	864 abA	960 abA	1029 aB	985 aA	1016 bB	1077 bB	987 aA	988 abA	1039 bB
	Romana	1047 abB	986 bA	1112 ab	1089 b	963 abA	1052 aB	1132 abB	1023 bA	1269 bB	1120 aA	1174 abB	1048 bA
	Wheat bran	944 ab	781 ab	1001 ab	786 ab	1117 ab	924 a	1179 ab	906 ab	1001 ab	790 a	1072 ab	849 ab
	Wheat flour	827 a	679 a	661 a	507 a	1010 ab	826 a	964 a	724 ab	791 a	603 a	881 ab	691 a
	Significance	***	**	***	**	**	*	*	*	***	*	*	*
8%	Achaak	1369 b	1394 bc	1306 bA	1427 bB	1209 bB	1192 abA	1203 bB	1175 aA	1365 bB	1196 abA	1427 bB	1280 abA
	Fakhfekh	979 abA	1430 bcB	1177 abA	1826 bB	858 abA	1527 abB	1043 abA	1447 abB	1319 bA	1687 bB	1232 bA	1505 bB
	Fascionello	1071 abA	1145 abB	1143 abB	1015 abA	1289 abB	782 aA	1290 bB	1022 aA	1208 abB	1119 ab	1143 bB	1016 abA
	Laurane	633 aA	1713 cB	663 aA	1822 bB	308 aA	1494 abB	698 aA	1556 bB	462 aA	1688 bB	351 aA	1400 bB
	Pizzuta	875 abA	1249 bB	948 abA	1395 bB	693 abA	1277 abB	739 aA	1320 abB	1002 abA	1200 abB	764 aA	1391 bB
	Romana	1265 bB	1078 abA	1413 bB	1112 abA	1142 abB	1023 abA	1069 ab	1107 a	1184 b	1364 bB	1046 bA	1110 abB
	Wheat bran	1218 ab	1283 b	958 ab	952 ab	1423 ab	1650 b	1223 b	1419 ab	899 ab	1002 ab	1350 b	1455 b
	Wheat flour	845 ab	858 a	646 a	602 a	1078 ab	1206 ab	988 ab	1123 a	816 ab	894 a	941 ab	993 a
	Significance	***	**	***	***	***	**	**	*	***	**	***	**

Sum of the ranks followed by different lowercase letters indicate significant difference at *p* < 0.05 among breads; sum of the ranks followed by different uppercase letters indicate significant difference at *p* < 0.05 for different harvesting times; *** *p* < 0.001; ** *p* < 0.01; * *p* < 0.05.

**Table 11:** Values (mean ± standard deviation; *n* = 3) of total phenolic content (TPC; mg GAE/100 g DW) and radical-scavenging activity (RSA; μmol TE/100 g DW) of the extracts obtained before and after in vitro digestion of breads prepared with 8% of Achaak hulls (green and mature), wheat bran, and wheat flour, and results of analysis of variance.

	TPC				RSA			
	Green	Mature	Wheat Bran	Wheat Flour	Green	Mature	Wheat Bran	Wheat Flour
Not digested	450.69 ± 8.29	294.74 ± 6.75	61.22 ± 1.05	52.95 ± 1.74	2204.49 ± 119.37	1498.20 ± 57.85	87.75 ± 4.90	30.69 ± 2.34
After in vitro digestion	272.89 ± 4.13	237.25 ± 5.98	23.71 ± 0.04	20.91 ± 0.48	1145.32 ± 33.16	750.12 ± 52.20	52.62 ± 1.45	24.32 ± 1.31
Significance	***	***	***	***	***	***	***	***

*** *p* < 0.001.

## Data Availability

Not applicable.
